# Correction of rhodopsin serial crystallography diffraction intensities for a lattice-translocation defect

**DOI:** 10.1107/S2059798323000931

**Published:** 2023-02-27

**Authors:** Matthew J. Rodrigues, Cecilia M. Casadei, Tobias Weinert, Valerie Panneels, Gebhard F. X. Schertler

**Affiliations:** a Laboratory of Biomolecular Research, Paul Scherrer Institute, 5232 Villigen PSI, Switzerland; b Department of Biology, ETH-Zurich, Zurich, Switzerland; University of Cambridge, United Kingdom

**Keywords:** serial crystallography, lattice-translocation defects, membrane proteins, lipidic cubic phase, G protein-coupled receptors

## Abstract

Rhodopsin is a G-protein-coupled receptor that detects light and initiates the intracellular signalling cascades that underpin vertebrate vision. Light sensitivity is achieved by covalent linkage to 11-*cis* retinal, which isomerizes upon photo-absorption. Serial femtosecond crystallography data collected from rhodopsin microcrystals grown in the lipidic cubic phase were used to solve the room-temperature structure of the receptor. Although the diffraction data showed high completeness and good consistency to 1.8 Å resolution, prominent electron-density features remained unaccounted for throughout the unit cell after model building and refinement. A deeper analysis of the diffraction intensities uncovered the presence of a lattice-translocation defect (LTD) within the crystals. The procedure followed to correct the diffraction intensities for this pathology enabled the building of an improved resting-state model. The correction was essential to both confidently model the structure of the unilluminated state and interpret the light-activated data collected after photo-excitation of the crystals. It is expected that similar cases of LTD will be observed in other serial crystallography experiments and that correction will be required in a variety of systems.

## Abstract 2

Correction of serial crystallography diffraction intensities for lattice-translocation defects enabled the interpretation of time-resolved serial femtosecond crystallography data probing the first molecular motions in vertebrate vision.

## 1. Introduction

The primary event in mammalian vision is the absorption of a photon by 11-*cis* retinal, which is covalently linked to a lysine side chain in rhodopsin via a protonated Schiff base (Nakanishi, 1991 ▸). Upon light absorption, retinal isomerizes to the all-*trans* form and the receptor transitions through a number of spectroscopically distinct intermediate states (Lewis & Kliger, 1992 ▸; Mathies & Lugtenburg, 2000 ▸). Once in the active state, the G protein-coupled receptor (GPCR) catalyses the exchange of GDP for GTP in the transducin G protein to initiate intracellular signalling cascades that result in neuronal signalling (Bennett *et al.*, 1982 ▸; Emeis *et al.*, 1982 ▸).

Structural and spectroscopic studies have exploited the abundance of rhodopsin in bovine retina to investigate how it performs its critical role in vision. The initial resting-state crystal structure of rhodopsin was followed by further crystal and cryo-EM structures of the receptor in the active state, in complex with signalling partners and in cryo-trapped intermediate states following light activation (Palczewski *et al.*, 2000 ▸; Standfuss *et al.*, 2011 ▸; Tsai *et al.*, 2018 ▸, 2019 ▸; Nakamichi & Okada, 2006*a* ▸,*b* ▸). However, until recently it has not been possible to determine how the structure of the protein changes as a function of time at physiological temperatures.

In the past decade, time-resolved serial femtosecond crystallography (TR-SFX) experiments have shone light on the photochemistry driving the activity of light-sensitive proteins (Poddar *et al.*, 2022 ▸). In particular, the molecular mechanisms of several retinal-dependent microbial opsins, including the proton pump bacteriorhodopsin (Nango *et al.*, 2016 ▸; Nogly *et al.*, 2018 ▸; Nass Kovacs *et al.*, 2019 ▸; Weinert *et al.*, 2019 ▸), the sodium pump KR2 (Skopintsev *et al.*, 2020 ▸), the chloride pump *Nm*HR (Yun *et al.*, 2021 ▸; Mous *et al.*, 2022 ▸) and the C1C2 channelrhodopsin cation channel (Oda *et al.*, 2021 ▸), have been deciphered. TR-SFX experiments with rhodopsins typically rely on the delivery of microcrystals embedded in a viscous medium to the interaction region (Nogly *et al.*, 2016 ▸; James *et al.*, 2019 ▸), where they are illuminated by an optical pulse. After a controlled time delay, the crystals are probed by an X-ray pulse from a free-electron laser (FEL). As only one, still diffraction image can be measured from an individual crystal before it is destroyed by the FEL X-ray pulse (Chapman *et al.*, 2014 ▸), a single data set is typically composed of images collected from tens of thousands of randomly oriented crystals.

While TR-SFX experiments are uniquely capable of visualizing protein conformational changes with atomic spatial and subpicosecond temporal resolution, the sample requirements are often challenging to fulfil. Crystallization in the lipidic cubic phase (LCP) has been the method of choice for most membrane proteins prepared for TR-SFX experiments (Landau & Rosenbusch, 1996 ▸), and LCP can also be used as a medium to deliver crystals to the X-ray beam via a high-viscosity sample injector (Weinert & Panneels, 2020 ▸).

Optimization of the bulk purification and LCP crystallization protocols for bovine rhodopsin yielded large quantities of well diffracting crystals that were suitable for time-resolved experiments at FELs (Wu *et al.*, 2015 ▸; Weinert & Panneels, 2020 ▸; Gruhl *et al.*, 2022 ▸).

It was possible to collect diffraction data sets from ‘dark-state’ crystals, which were not illuminated, and also from crystals 1, 10 and 100 ps after light activation at the Swiss Free Electron Laser (SwissFEL) and the SPring-8 Angstrom Compact Free-Electron Laser (SACLA) (Gruhl *et al.*, 2022 ▸; Table 1 ▸). An initial model of the dark-state structure could be obtained by molecular replacement, after which several iterations of model building and refinement were carried out. Inspection of the electron-density maps revealed significant density features in the solvent channels that could not be successfully modelled by placing solvent molecules.

After in-depth analysis of the electron-density maps and diffraction intensities, we were able to identify the presence of a lattice-translocation defect within the rhodopsin crystals. Procedures to correct rotation data collected from single crystals exhibiting such defects have been described previously (Wang, Kamtekar *et al.*, 2005 ▸; Wang, Rho *et al.*, 2005 ▸). Here, we describe the procedure performed to detect, characterize and correct serial crystallography data sets for this pathology. The correction was essential to confidently model both the dark and light-activated structures of rhodopsin, and therefore to elucidate the first molecular rearrangements in vertebrate vision.

## 2. Data collection and initial processing

### 2.1. Data collection

Bovine rhodopsin was purified from the native source and crystallized in LCP as has been described in detail (Gruhl *et al.*, 2022 ▸). The microcrystals embedded in LCP were delivered to the X-ray beam by a high-viscosity sample injector (Weierstall *et al.*, 2014 ▸) and diffraction data were collected from crystals either without illumination by the pump laser (dark sample) or at a defined time delay after illumination. Data were collected as still images at SwissFEL and SACLA, with separate dark-state data sets measured at both FELs. Experimental parameters are detailed in Gruhl *et al.* (2022 ▸).

### 2.2. Initial processing

As described in Gruhl *et al.* (2022 ▸), all data collected at SwissFEL (SF dark, 1 ps, 10 ps) were indexed using *indexamajig* (White *et al.*, 2012 ▸) with the *XGANDALF* algorithm (Gevorkov *et al.*, 2019 ▸). The *MOSFLM*, *DirAx* and *XGANDALF* methods (Powell, 1999 ▸; Duisenberg, 1992 ▸; Gevorkov *et al.*, 2019 ▸) were used for the data collected at SACLA (SACLA dark, 100 ps). Diffraction images collected at SwissFEL were indexed in space group *P*22_1_2_1_ with unit-cell parameters *a* = 61.51, *b* = 91.01, *c* = 151.11 Å and data collected at SACLA were indexed in the same space group with unit-cell parameters *a* = 61.29, *b* = 90.81, *c* = 150.51 Å.

All data were integrated using *indexamajig* in *CrystFEL* (White *et al.*, 2012 ▸). The integration radius was set to two pixels for SwissFEL data and three pixels for SACLA data, while the background annulus was set to between four and six pixels for SwissFEL data and between four and seven pixels for data collected at SACLA. The crystal-to-detector distance was optimized by sampling detector distances between 91.5 and 97.5 mm at SwissFEL and 47.5 and 53.5 mm at SACLA first in 200 µm increments and then in 20 µm increments to determine the detector distance at which the standard deviations of the unit-cell parameters were minimized.

Initial phasing by molecular replacement was performed with *Phaser* (McCoy *et al.*, 2007 ▸) using a deposited structure of rhodopsin at cryogenic temperature (PDB entry 1u19; Okada *et al.*, 2004 ▸), with solvent and ligand molecules removed, as a search model. The asymmetric unit contains two rhodopsin molecules arranged as an antiparallel dimer. Rotation of this antiparallel dimer around the crystallographic screw axes generates translational noncrystallographic symmetry (tNCS), which was detected as a peak with a magnitude 65% of the origin peak height at position 0.5**a** + 0.378**b** + 0.5**c** in the native Patterson map with *phenix.xtriage* (Liebschner *et al.*, 2019 ▸). Multiple iterations of model building in *Coot* (Casañal *et al.*, 2020 ▸) and refinement with *phenix.refine* (Liebschner *et al.*, 2019 ▸) were carried out until convergence of the *R*_free_ statistics. Most metrics of model quality were reasonable at this stage (see Tables 2 ▸ and 3 ▸). In particular, model refinement against dark-state data collected at SwissFEL yielded *R*_work_ and *R*_free_ values of 26.65% and 28.27%, respectively, in the resolution range 16.1–1.8 Å, showing that the modelled structure explained the experimental observations well.

## 3. Indications of lattice-translocation disorder in real space

Although the *R*-factor statistics, which quantify the agreement between the observed and model structure factors, converged to values which are commonly considered to be acceptable in serial crystallography, inspection of the electron-density maps indicated that our model did not appropriately describe the underlying data. As is common practice, we calculated σ_A_-weighted electron-density difference maps, where the Fourier coefficients (*mF*_o_ − *DF*_c_)exp(*i*φ_c_), with observed structure-factor amplitudes *F*_o_ and model amplitudes and phases *F*_c_, φ_c_, include weighting factors *m* and *D* to account for the coordinate errors within the model and the partiality of the modelled structure (Read, 1986 ▸). These maps are less affected by model bias compared with (*F*_o_ − *F*_c_)exp(*i*φ_c_) maps and are particularly useful to identify errors in the model and aid model building. In particular, positive difference peaks correspond to features of the electron density which are unaccounted for by the model.

Significant positive difference electron-density features were visible both in the solvent channels between rhodopsin molecules and overlapping with the rhodopsin dimer (Fig. 1 ▸). This difference density could not be sensibly modelled by the placement of solvent molecules. Furthermore, some of the observed features resembled amino-acid side chains (Fig. 2 ▸ *a*). However, there was insufficient space between the protein molecules to accommodate an additional rhodopsin chain.

We were able to model a single tryptophan residue into a particularly prominent positive difference electron-density feature in the solvent channel (Fig. 2 ▸ *a*) and then to least-squares align tryptophan-centred tripeptides from the rhodopsin structure to the placed tryptophan residue in *Coot* (Fig. 2 ▸ *b*). In this way, we could assign this unexplained density feature to Trp265. Least-squares alignment of the entire rhodopsin dimer to this tryptophan residue resulted in the model overlapping well with the unexplained density (Fig. 2 ▸ *c*). This second copy of the dimer was related to the original structure by a translation of 22.5 Å along the unit-cell **b** axis, corresponding to approximately one-quarter of the length of the **b** axis. As the original dimer and the translated copy spatially overlap, it is not possible for both copies to be present in the same unit cell.

This observation was reminiscent of several previous observations of lattice-translocation disorder (LTD), namely the presence of translation-related domains in the crystals, in a number of systems (Bragg & Howells, 1954 ▸; Pickersgill, 1987 ▸; Wang, Rho *et al.*, 2005 ▸; Hare *et al.*, 2009 ▸; Tsai *et al.*, 2009 ▸; Ponnusamy *et al.*, 2014 ▸; Li *et al.*, 2020 ▸). We therefore sought to determine whether this was another such case and whether the data could be corrected as previously described in Wang, Kamtekar *et al.* (2005 ▸).

As a first step, a Fourier transform of the merged intensities was performed to produce a Patterson map, which represents the autocorrelation function of the electron density in real space and exhibits prominent peaks at positions corresponding to highly frequent interatomic and intermolecular vectors in the structure. By inspecting this map, we observed a prominent peak at the position 0.5**a** + 0.378**b** + 0.5**c**, where **a**, **b**, **c** are the unit-cell axes, which was accounted for by the noncrystallographic symmetry operation relating the two copies of the molecule in the asymmetric unit. We also observed a second prominent peak, with a magnitude of 18% of the origin peak for the SACLA data and 25% of the origin peak for the SwissFEL data, at 0.245**b** (Fig. 3 ▸). We attributed this peak to the vector **t**_d_ relating the two translation-related domains.

## 4. Indications of lattice-translocation disorder in reciprocal space

We assume that terms from translation-related domains corresponding to integer multiples of the fundamental translation **t**_d_ contribute to the structure factors of an LTD-affected crystal. Following and generalizing the treatment of LTD presented in Wang, Kamtekar *et al.* (2005 ▸), we model such structure factors with the weighted sum of an infinite number of translation-related terms, 

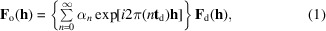

where **h** denotes the reciprocal-lattice point *h* a* + *k* b* + *l* c* with integer *h*, *k*, *l* and reciprocal-lattice basis vectors a*, b*, c*. Real-space domain translations are given by the set {*n* **t**_d_} of integer multiples of the fundamental translation **t**_d_, and the weights α_*n*_ represent the unit-cell fractions pertaining to each domain. The single-domain structure factor **F**_d_ is given by the usual sum over contributions from all atoms within one unit cell, 

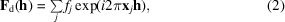

where *f*_*j*_ is the atomic form factor and **x**_*j*_ is the position of atom *j* in the unit cell. The structure factor of a translated domain is 

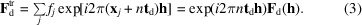




Inspection of the Patterson map provides the estimate **t**_d_ = *t*_d_ **b** = 0.245**b**. Because high-order terms are progressively less relevant, we truncate the summation in equation (1) to the term with *n* = 2, obtaining 

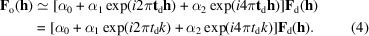

As a result of squaring the complex structure factors to obtain diffraction intensities 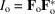 , interference terms (α_0_α_1_ + α_1_α_2_)[exp(i2π*t*_d_ *k*) + exp(−*i*2π*t*_d_ *k*)] and α_0_α_2_[exp(*i*4π*t*_d_ *k*) + exp(−*i*4π*t*_d_ *k*)] appear. This gives rise to a characteristic oscillatory behaviour of the observed intensities as a function of reciprocal space index *k*, 

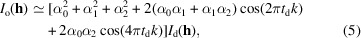

with 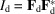 . This oscillatory behaviour can be recognized in Fig. 4 ▸, where measured intensities, averaged over reciprocal-space indices (*h*, *l*), are shown as a function of index *k*.

## 5. Correction of diffraction intensities

The inspection and analysis of the electron-density maps, the Patterson map and the merged intensities supported the diagnosis of LTD within the rhodopsin crystals. Tentative domain fractions α_0_, α_1_ and α_2_ were sampled with steps of 0.01 in the range [0, 1]. For each triplet (α_0_, α_1_, α_2_) satisfying 

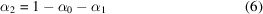

with 

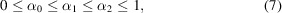

we estimated individual domain intensities by inversion of equation (5) as follows: 

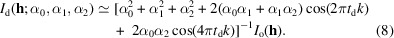




For each set of corrected intensities *I*_d_(**h**; α_0_, α_1_, α_2_), we calculated the Patterson map value at positions *t*_d_ **b** and 2*t*_d_ **b**. The values of 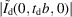 and 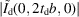 , where 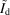 is the Fourier transform of *I*_d_, are reported in Figs. 5 ▸ and 6 ▸ for the SwissFEL and SACLA data, respectively, as a function of (α_0_, α_1_). We optimized the domain fractions by selecting the triplet resulting in small Patterson peaks at both examined positions. We obtained α_0_ = 0.01, α_1_ = 0.19 and α_2_ = 0.80 for the SwissFEL data set and α_0_ = 0.00, α_1_ = 0.15 and α_2_ = 0.85 for the SACLA data set. Fig. 3 ▸ shows a comparison of the Patterson map values before and after correction along the direction of the unit-cell axis **b**. Upon correction of the intensities using the optimized values, a dampening of the oscillations of (*h*, *l*)-averaged values as a function of index *k* was observed (Fig. 4 ▸).

With the definitions

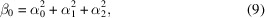




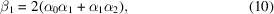

and 

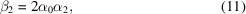

equation (5) can be rewritten 

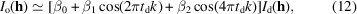

with weights β_0_ = 0.6762, β_1_ = 0.3078, β_2_ = 0.016 for the SwissFEL data and β_0_ = 0.745, β_1_ = 0.255, β_2_ = 0 for the SACLA data. The ratios β_1_/β_0_ (0.46 and 0.34 for the SwissFEL and SACLA data, respectively) and β_2_/β_0_ (0.02 and 0 for the SwissFEL and SACLA data, respectively) express the relative importance of interference terms for increasing relative displacement and show that it is well justified to neglect terms with *n* > 2 in equation (1).

Translation correction factors were determined separately for each of the data sets. Refinement of the rhodopsin dark-state atomic model against the LTD-corrected intensities from SwissFEL yielded an immediate improvement in both the *R*_work_ and *R*_free_ statistics (21.43% and 24.61%, respectively) and the model geometry. More importantly, the amount of unexplained *mF*_o_ − *DF*_c_ difference density was much reduced in the resulting electron-density maps. This allowed further model building to remove inappropriately placed solvent molecules, fix distorted amino-acid side-chain rotamers and build more of the flexible loops in the protein. The dark-state final models therefore accounted better for the experimental data. Improved model phases were available for calculation of the light-to-dark electron-density difference maps, with Fourier coefficients 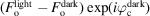 , derived from the observed amplitudes 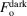 and 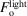 of the dark and light-activated state, respectively, and dark model phases 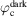 . These maps are essential to interpret the conformational changes following light activation.

There is a stark contrast between the *mF*_o_ − *DF*_c_ maps produced after refinement of the final dark-state model against the diffraction intensities with and without correction, demonstrating the importance of the correction in obtaining an accurate dark-state model (Figs. 7 ▸ and 8 ▸). As the dark-state model serves as the foundation for interpreting light-induced structural changes, the correction described was required to confidently derive biological insights into the very first processes in receptor activation (Gruhl *et al.*, 2022 ▸).

## 6. Discussion

Several examples of LTD in crystals of soluble proteins have been published since the first observation in 1954 (Bragg & Howells, 1954 ▸). Furthermore, occurrences of LTD may be underreported, as this pathology is difficult to detect and may often be ignored when the translated fraction is small (Lovelace & Borgstahl, 2020 ▸). LTD has also been identified in LCP-grown crystals of the rice silicon transporter Lsi1 (Saitoh *et al.*, 2021 ▸). Similar to our observation of LTD in rhodopsin crystals, the Lsi1 lattice translation appears to occur parallel to the plane of the membrane. Indeed, lattice translations perpendicular to the plane of the LCP bilayer seem improbable as the hydrophobic transmembrane region of the protein would be displaced from the hydrophobic lipid environment to the aqueous environment between the lipid bilayers. Instead, type I LCP crystals, which assemble as stacks of 2D crystals, appear to be susceptible to translations in the relative positions of these 2D layers, which result in translations parallel to the plane of the membrane.

In the previously observed cases of LTD the data were collected using the rotation method (Dauter, 1999 ▸) and it was possible to diagnose the disorder by direct inspection of the diffraction images. In Wang, Kamtekar *et al.* (2005 ▸), for example, a translation of 0.5 in the **c** axis in Bacillus phage φ29 DNA polymerase crystals resulted in the appearance of weak and streaky spots for reflections with odd values of *l* and strong spots for even values of *l*, caused by the main lattice and the translated lattice scattering out of phase and in phase with odd and even values of *l*, respectively.

Such an analysis is not possible with serial crystallography data, as the measured intensities from individual frames are partial and the intensity of a given reflection cannot be determined by inspection of a single diffraction image. Instead, it is necessary to search for non-origin peaks in the Patterson map that are too close to the origin to be explained by tNCS.

Despite working with a single protein purification and crystallization protocol, we observed some variability in the determined values of the translated domain fractions depending on the examined data set. While data sets from the SwissFEL beamtime showed α_0_ and α_1_ values close to 0.01 and 0.19, respectively, we found that data sets collected during a previous beam time at SACLA had lower translated domain fractions, with values of α_0_ = 0.00 and α_1_ = 0.15. The unexplained difference density was therefore less prominent after model refinement using uncorrected intensities from the SACLA data set. Although the crystalline sample was prepared using the same protocol for both beam times, subtle differences in the sample preparation such as the crystallization temperature or precipitant composition may affect the probability of a lattice translocation occurring during crystal growth. Such a variation in the translated fraction has previously been observed in crystals of lentiviral integrase in complex with LEDGF and was ascribed to differences in crystal-growth conditions (Hare *et al.*, 2009 ▸). It is also possible that the smaller beam size used at SACLA, 1 × 1 µm compared with 5 × 5 µm at SwissFEL, reduced the chance of collecting data from a region of the crystal containing a lattice-translocation defect.

The translated domain fractions were found to be similar for all data sets collected during the same beam time. This is to be expected as both dark and light data sets were collected from the same sample batches and the translated domain fractions are the result of an average over all diffracting crystals contributing to each data set.

We attempted the refinement of a composite atomic model containing the main dimer at occupancy (1 − κ) and a second model of the dimer with occupancy κ, translated from the main model by **t**_d_, against the uncorrected data, as was previously successful for the 1918 H1N1 neuraminidase (Zhu *et al.*, 2008 ▸). The introduction of an additional copy of the dimer during refinement with our serial crystallography rhodopsin data necessitates the use of strict NCS constraints to avoid significantly worsening the data-to-parameter ratio. However, the refinement of a composite model against uncorrected data was deemed impractical with bovine rhodopsin, as interpretation of the 2*mF*_o_ − *DF*_c_ electron-density maps was challenging in the region where the two models overlapped. As previously observed by Zhu *et al.* (2008 ▸), we found that correction of the data for LTD was required to model solvent molecules in the overlapped region accurately. The positions of ordered water molecules are of key interest in studies of receptor dynamics as they often stabilize hydrogen-bond networks in the transitions between conformational states.

We did not observe dramatic changes in the time-resolved 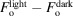 difference maps as a result of the correction (Fig. 9 ▸). This is because the light and dark data sets were collected from the same sample using an interleaved sequence of visible light laser pulses to pump the sample and X-rays to probe the structure (Nango *et al.*, 2019 ▸). The systematic error due to LTD is therefore very similar in both data sets, usually with less than 1% difference in the translated populations. Instead, the most significant effect of LTD was to increase the number of poorly modelled regions in the dark-state models. As the dark-state model was taken as a starting point for the refinement of light-state structures into the extrapolated maps generated from the light-activated data sets, correction for LTD improved our models of both the dark and light-activated rhodopsin structures.

## 7. Conclusions

TR-SFX experiments provide unprecedented opportunities to understand ultrafast biological processes, including the photo-isomerization of retinal in rhodopsin, which is the first event in mammalian vision. Having overcome many of the challenging barriers to performing a TR-SFX experiment with rhodopsin, we identified the LTD in the crystals at the data-processing stage. The described correction facilitated the interpretation of the data collected during these time- and resource-intensive experiments (Gruhl *et al.*, 2022 ▸). Given the demanding pressures on sample preparation and beam time, it is often not possible to repeat an experiment after optimization of the crystals to avoid the LTD.

Here, we show the identification and characterization of LTD in a serial crystallography experiment. As these experiments, including time-resolved studies, become more widespread, it is likely that the present method of LTD correction will be of choice. 

## Supplementary Material

PDB reference: bovine rhodopsin in lipidic cubic phase, dark state, SACLA, 7zbc

PDB reference: dark state, SwissFEL, 7zbe

PDB reference: 1 ps light-activated, 8a6c

## Figures and Tables

**Figure 1:**
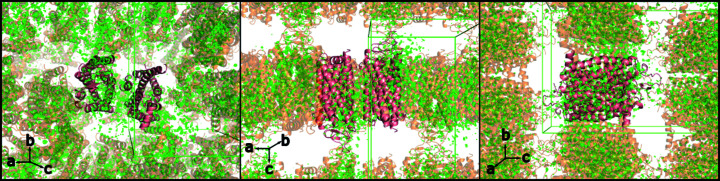
Rhodopsin dimer (pink) and symmetry-related rhodopsin molecules (pale orange) shown in cartoon format with σ_A_-weighted *F*_o_ − *F*_c_ difference density contoured at 4.0σ overlaid (green). The unit cell is shown as a green box.

**Figure 2:**
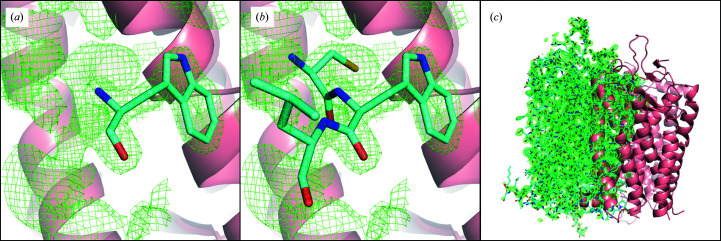
Rhodopsin dimer in cartoon format (pink) overlaid with σ_A_-weighted *F*_o_ − *F*_c_ difference density contoured at 2.0σ (green mesh). (*a*) Trp265 (cyan) fitted into σ_A_-weighted *F*_o_ − *F*_c_ difference density. (*b*) Cys264, Trp265 and Leu266 (cyan) aligned to the fitted Trp265. Transmembrane helices 5 (left) and 6 (right) are visible behind the translated peptide. (*c*) A translated rhodopsin dimer (cyan stick format) aligned with Trp265 overlaid with the main rhodopsin dimer.

**Figure 3:**
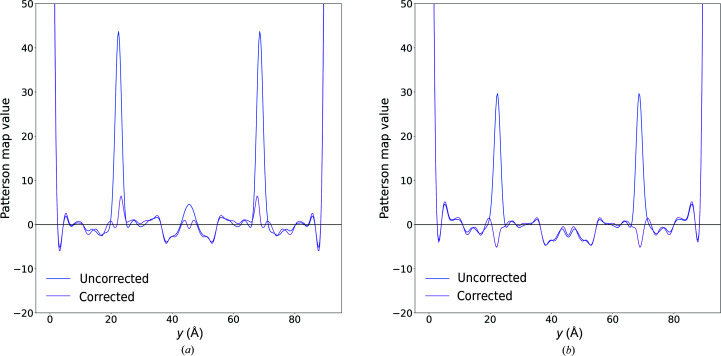
Patterson map values for SwissFEL data (*a*) and SACLA data (*b*) along the direction 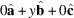 , where 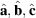 are unit vectors aligned with the corresponding unit-cell basis vectors before and after correction of the intensities with α_0_ = 0.01, α_1_ = 0.19, α_2_ = 0.80 (SwissFEL) and α_0_ = 0.00, α_1_ = 0.15, α_2_ = 0.85 (SACLA). The peak height at 0.245**b** is equivalent to 25% and 18% of the origin peak heights for uncorrected SwissFEL and SACLA data, respectively.

**Figure 4:**
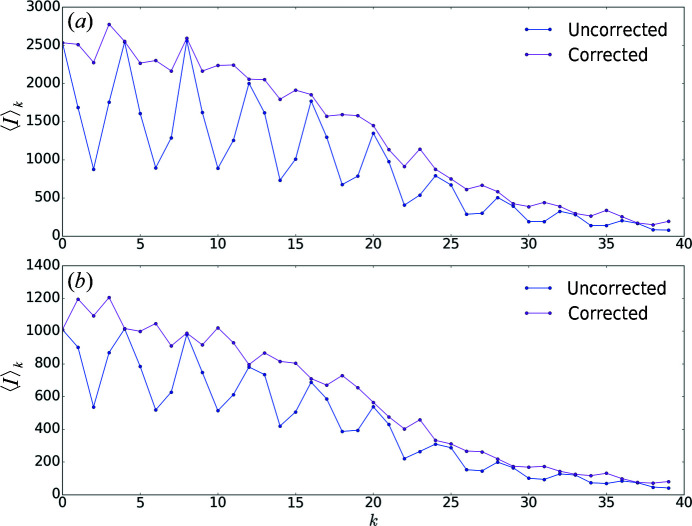
Diffraction intensities from SwissFEL (*a*) and SACLA (*b*), averaged over reciprocal-space indices *h* and *l*, as a function of index *k* before and after correction with α_0_ = 0.01, α_1_ = 0.19, α_2_ = 0.80 (SwissFEL) and α_0_ = 0.00, α_1_ = 0.15, α_2_ = 0.85 (SACLA).

**Figure 5:**
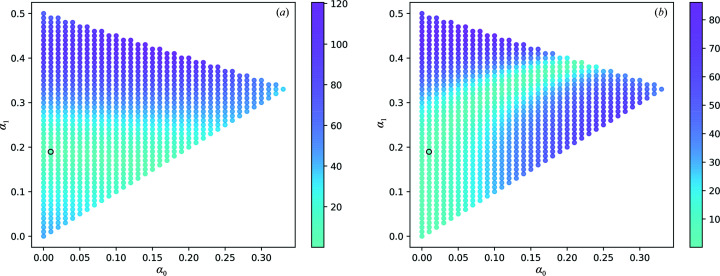
Absolute values of the Patterson function of corrected SwissFEL intensities with varying α_0_, α_1_ and α_2_ = 1 − α_0_ − α_1_ at real-space positions *t*_d_ **b** (*a*) and 2*t*_d_ **b** (*b*). Correction of intensities with α_0_ = 0.01, α_1_ = 0.19 (black circle) dampens the Patterson peaks at both positions simultaneously.

**Figure 6:**
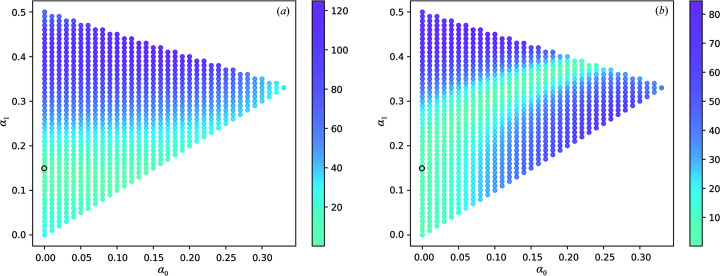
Absolute values of the Patterson function of corrected SACLA intensities with varying α_0_, α_1_ and α_2_ = 1 − α_0_ − α_1_ at real-space positions *t*_d_ **b** (*a*) and 2*t*_d_ **b** (*b*). Correction of intensities with α_0_ = 0.00, α_1_ = 0.15 (black circle) dampens the Patterson peaks at both positions simultaneously.

**Figure 7:**
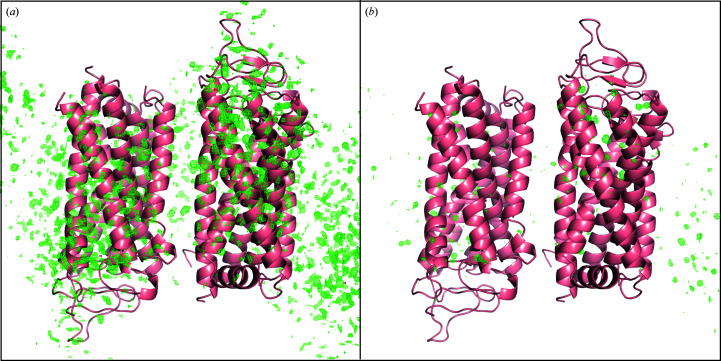
Map showing *mF*_o_ − *DF*_c_ difference electron density after refinement of the SwissFEL dark-state model against (*a*) the original diffraction intensities (contoured at 0.514 e^−^ Å^−3^, equivalent to 4.00σ) and (*b*) the corrected intensities (contoured at 0.514 e^−^ Å^−3^, equivalent to 4.96σ).

**Figure 8:**
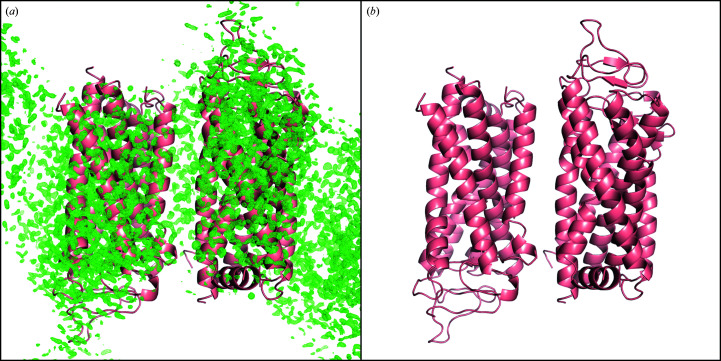
Map showing *mF*_o_ − *DF*_c_ difference electron density after refinement of the SACLA dark-state model against (*a*) the original diffraction intensities (contoured at 1.071 e^−^ Å^−3^, equivalent to 4.00σ) and (*b*) the corrected intensities (contoured at 1.071 e^−^ Å^−3^, equivalent to 11.04σ).

**Figure 9:**
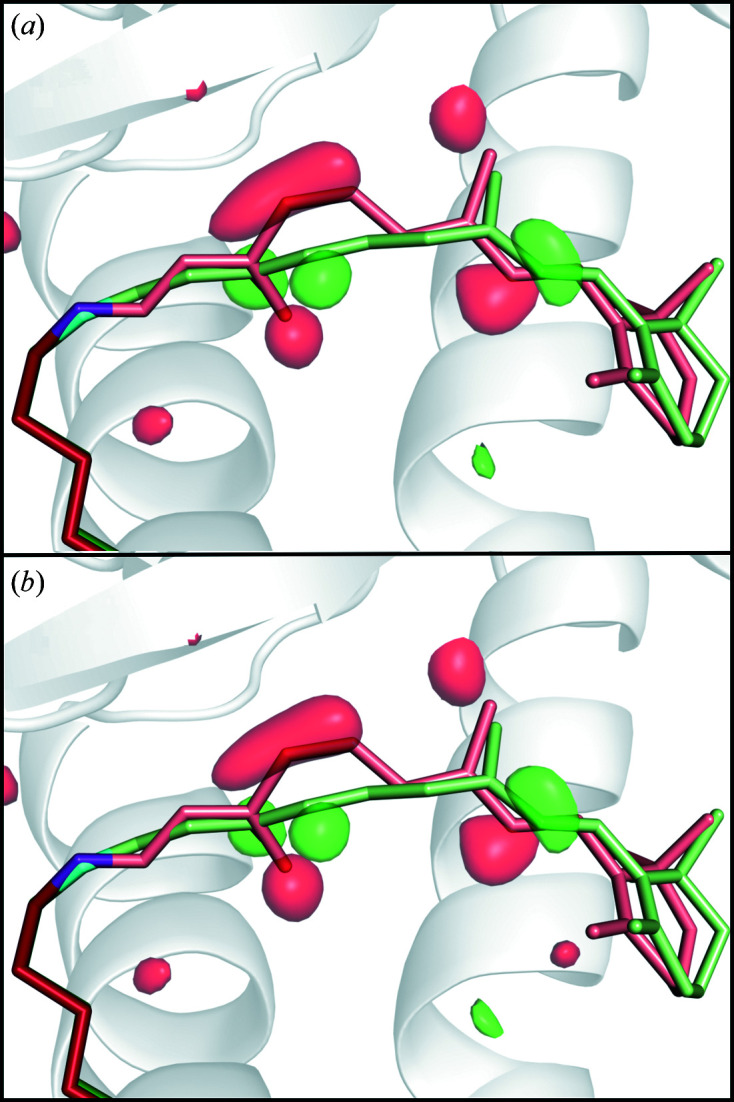
Isomorphous 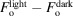 difference electron density within 3.2 Å of the retinal chromophore 1 ps after light activation. Dark-state (SwissFEL) C atoms of Lys296 are shown in red, N atoms in purple and retinal atoms in pink. 1 ps light-activated (SwissFEL) C atoms of Lys296 are shown in dark green, N atoms in cyan and retinal atoms in light green. (*a*) Original diffraction intensities (contoured at 4.0σ, equivalent to 0.075 e^−^ Å^−3^) and (*b*) corrected intensities (contoured at 4.0σ, equivalent to 0.075 e^−^ Å^−3^).

**Table 1:** Merging statistics for dark-state data sets collected at SwissFEL and SACLA (Gruhl *et al.*, 2022 ▸) Values in parentheses are for the highest resolution shell.

Data set	SwissFEL	SACLA
Resolution range (Å)	16.10–1.80 (1.86–1.80)	10.47–1.80 (1.86–1.80)
*a*, *b*, *c* (Å)	61.51, 91.01, 151.11	61.29, 90.81, 150.51
α, β, γ (°)	90.0, 90.0, 90.0	90.0, 90.0, 90.0
Space group	*P*22_1_2_1_	*P*22_1_2_1_
Measured reflections	65870940 (4371475)	105018485 (7436654)
Unique reflections	79305 (7852)	78209 (7715)
Multiplicity	830.6 (556.7)	1342.8 (963.9)
Completeness (%)	100.0 (100.0)	100.0 (100.0)
〈*I*/σ(*I*)〉	7.62 (0.95)	8.87 (1.32)
*R*_split_ (%)	8.21 (109.06)	6.75 (77.70)
CC*	0.9982 (0.8947)	0.9994 (0.9219)
CC_1/2_	0.9926 (0.6672)	0.9977 (0.7391)
Translation vector (**t**_d_)	0.245	0.243
Translated fraction	α_0_ = 0.01, α_1_ = 0.19	α_0_ = 0.00, α_1_ = 0.15

**Table 2:** Refinement statistics for the SwissFEL dark-state model refined against the original and the corrected diffraction intensities

	Original intensities	Corrected intensities
*R*_work_/*R*_free_ (%)	26.65/28.27	21.43/24.61
No. of atoms		
Protein	4971	4971
Ligand	526	526
Water	174	174
*B* factors (Å^2^)		
Protein	31.60	32.20
Ligand	46.77	49.08
Water	38.20	40.23
Ramachandran statistics		
Favoured (%)	96.48	96.81
Allowed (%)	3.52	3.19
Outliers (%)	0.00	0.00
R.m.s. deviations		
Bond angles (°)	0.009	0.007
Bond lengths (Å)	0.928	0.847

**Table 3:** Refinement statistics for the SACLA dark-state model refined against the original and the corrected diffraction intensities

	Original intensities	Corrected intensities
*R*_work_/*R*_free_ (%)	22.38/23.68	19.50/22.11
No. of atoms		
Protein	4970	4970
Ligand	539	539
Water	168	168
*B* factors (Å^2^)		
Protein	30.48	31.16
Ligand	47.53	49.74
Water	38.13	39.88
Ramachandran statistics		
Favoured (%)	96.64	97.14
Allowed (%)	3.36	2.86
Outliers (%)	0.00	0.00
R.m.s. deviations		
Bond angles (°)	0.005	0.009
Bond lengths (Å)	0.738	0.977
